# Preschool Asthma Symptoms in Children Born Preterm: The Relevance of Lung Function in Infancy

**DOI:** 10.3390/jcm9103345

**Published:** 2020-10-18

**Authors:** Manuel Sanchez-Solis, Maria Soledad Parra-Carrillo, Pedro Mondejar-Lopez, Patricia W Garcia-Marcos, Luis Garcia-Marcos

**Affiliations:** 1 Pediatric Pulmonology Unit, Virgen de la Arrixaca University Hospital, Murcia University, 30003 Murcia, Spain; mondejarp@gmail.com (P.M.-L.); part.garcia.marcos@gmail.com (P.W.G.-M.); lgmarcos@um.es (L.G.-M.); 2 Department of Surgery, Pediatrics, Obstetrics and Gynecology, University of Murcia, 30003 Murcia, Spain; mariasoledadparrac@gmail.com; 3 Arrixaca Bioresearch Institute of Murcia, 30003 Murcia, Spain

**Keywords:** pre-school asthma, preterm newborn, infant lung function

## Abstract

**Background:** The aim of the study is to assess whether lung function of infants born preterm predicts wheezing in pre-school age. **Methods:** A survey of the core wheezing questionnaire of the International Study on Asthma and Allergy in Children was administered to parents of preterm newborns, to whom lung function tests were performed at a corrected age of six months, and who, at the time of the survey, were between three and nine years of age. **Results:** Low values of all lung function parameters measured, except FVC, were predictors of wheezing at some time in life, (FEV0.5 OR: 0.62 (95%CI 0.39; 0.995); FEV0.5/FVC OR: 0.73 (0.54; 0.99)) FEF75 OR: 0.60 [0.37; 0.93]; FEF25-75 OR: 0.57 (0.37; 0.89)); and of wheezing in the past year (FEV0.5 OR: 0.36 (0.17; 0.76); FEV0.5/FVC OR: 0.59 (0.38; 0.93); FEF75 OR: 0.38 [0.19; 0.76]; FEF25-75 OR: 0.35 (0.17; 0.70). In addition, FEV0.5/FVC values lower than the lowest limit of normality, were predictive of hospital admissions due to wheezing (OR: 3.07; (1.02; 9.25)). **Conclusions:** Limited lung function in infancy is predictive of both future wheezing and hospitalization for a wheezing episode.

## 1. Introduction

According to previous studies, children born preterm have an increased risk of wheeze and asthma as compared to children born at term. This risk is higher when gestational age is lower [1] and is independent of a family history of atopy [2]. Additionally, preterm children have lower lung function during their infancy than their counterparts born at term [3,4] and this low lung function persists until school age [5,6] and even into adulthood [7,8].

Different perinatal factors have also been related with subsequent respiratory morbidity in children born preterm; including bronchopulmonary dysplasia (BPD) [5,6,8,9,10], intrauterine growth restriction [10,11], male sex [10] and Afro-American ethnicity [10], as well as non-perinatal factors: mainly, lower lung function, generally expressed in lower values of forced expiratory volume in the first second (FEV1), at the age when the study was performed [6].

One study [12] on the differences in lung function between infants with BPD and healthy ones, showed that the infants with BDP who presented recurrent wheeze had significant reductions in forced expiratory flow at 25% of the forced vital capacity (FEF25), and increased residual volume (RV), as compared to the normal infants. Furthermore, even when they were compared to their counterparts without wheeze, those infants who had suffered BPD and wheeze had both RV as well as the ratio between RV and the total lung capacity (TLC) significantly higher.

On the other hand, at least three cohorts that recruited healthy newborns to study risk factors for asthma inception have found that low lung function in infants is a risk factor for subsequent asthma [13,14,15], which suggests a very early origin of the condition; and that pre- and peri-natal factors must play an important role.

To the best of our knowledge, the relationship between lung function in preterm infants and respiratory morbidity at later ages has not been previously studied. Our objective is to study if, among children born prematurely, low values of different lung function parameters measured in infancy are risk factors for wheezing at pre-school age.

## 2. Experimental Section

### Methods

Study population: This is a retrospective study, carried out on ex-preterm newborns, (gestational age under 32 weeks), to whom lung function tests were administered at a corrected age of six months and who, at the time of a telephone survey were between three and nine years of age. Children were born between 2010 and 2016. The initial sample included 167 patients, of whom a total of 142 accepted to participate. Patients who required oxygen beyond postmenstrual week 36 were diagnosed.

Telephone survey: Parents of the patients answered, over the telephone, the core wheezing questionnaire of the International Study on Asthma and Allergy in Children (ISAAC) http://isaac.auckland.ac.nz/resources/tools.php?menu=tools1), validated internationally.

Infant lung function: The forced vital capacity (FVC), the forced expiratory volume at 0.5 sec (FEV_0.5_), the forced expiratory flows at 75% and between 25%–75% of the FVC (FEF_75_ and FEF_25-75_, respectively) and the ratio of FEV_0.5_/FVC were measured at a corrected mean age of 27.7 ± 1.94 weeks, from the maximum expiratory curves obtained by means of the Rapid Chest Compression with Pre-insufflation technique in accordance with the American Thoracic Society and the European Respiratory Society (ATS-ERS) recommendations [16]. The tests were performed with Master-Screen Baby Body Plethysmograph equipment (Jaëger^®^, Germany) and the pre-insufflation was by means of coupling the Neopuff neonatal resuscitator (Fisher & Paykel Healthcare^®^, New Zealand) with the face mask. All the infants were sedated with chloral hydrate at a dose of 80–100 mg/Kg and oxygen saturation was continually controlled using pulse oximetry.

Rehospitalizations due to respiratory causes: the number of admissions to hospital due to respiratory causes was obtained from review of the “Ágora” computer register of the Health Authority of Murcia, which records all admissions and their causes for all the population of the Region of Murcia, Spain. The policy regarding the use of Palivizumab, in our medium, is to administer it on the first day of life to all patients with BPD and to all the preterm newborns below 29 weeks of gestational age; thus, almost all the sample (96%) was treated.

Statistical study: To calculate the power of the study, a variance in the z-score of FEV_0.5_ (0.76) found in this study has been considered. For a difference in the mean FEV_0.5_ z-score of 0.5, a 95% confidence interval, and a power of ≥80%, required a total of 48 patients per group.

The association between qualitative variables was tested by means of the analysis of the contingency tables using Pearson’s Chi-squared statistic. Student’s t-test was used to compare the means of the quantitative variables.

Additionally, multivariate regression logistic analyses were carried out, with the presence of wheezing in the past (yes/no) being the dependent variable and including the following independent variables: age at time of survey; sex; attending day-care/school (yes/no); presence of BPD; gestational age (weeks); birthweight z-score; allergic mother (yes/no); and z-score of each of the lung function parameters (FVC, FEV_0.5_, FEV_0.5_/FVC, FEF_75_ and FEF_25-75_) in different models: one model for each lung function parameter. The same analyses were performed considering exclusively wheeze in the past year (yes/no) as the dependent variable and with the same independent variables.

On the other hand, linear regression analyses were carried out, using the number of wheezing episodes as the dependent variable. The following were used as independent variables: age at time of survey; sex; attending day-care/school (yes/no); presence of BPD; gestational age (weeks); birthweight z-score; and allergic mother (yes/no). As in the previous case, the analyses were repeated including, as the independent variable, each of the results of the z-score of the lung function parameters, with a different model for each one.

The study was approved by the Ethics Committee of the Virgen de la Arrixaca University Hospital from Murcia (ethical approval code: 2007-1-1-HCUVA).

## 3. Results

From the initial sample, which included 167 patients, a total of 142 (85.03%) accepted to participate.

### 3.1. Wheezing Ever

Some 50% of the children studied presented wheeze at some point on their life (Table 1).

**Figure 1:**
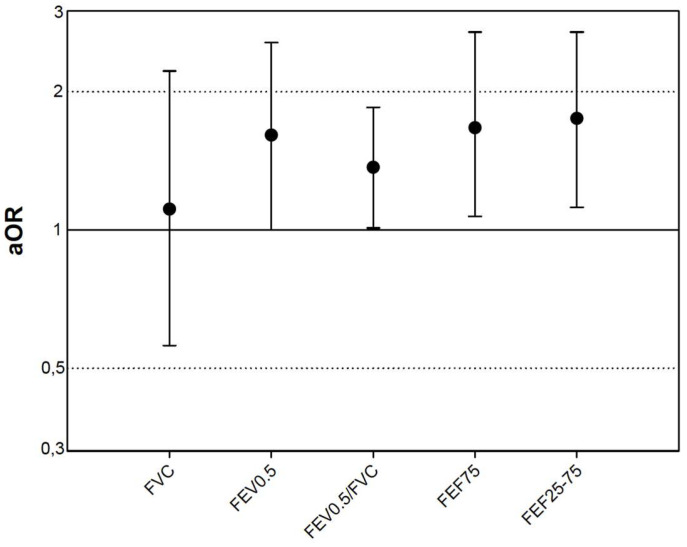
Risk of wheezing ever per z-score unit decrease of lung function. Adjusted odds ratios corrected for age, gender, daycare attendance, gestational age, birth weight (z-score), bronchopulmonary dysplasia, and allergic mother.

The univariate analysis showed that the presence of wheezing is associated with the z-score of different lung function measurements (FEV0.5, FEF75 and FEF25-75). The diagnosis of BPD constituted a consistent risk factor in all the multivariate analyses, independently of the lung function parameter included in it, with the OR value ranging from 2.45 (95% CI 1.07–5.63), *p* = 0.03 to 2.32 (95% CI 1.01–5.35), *p* = 0.047. Likewise, the mother having allergy also constituted a consistent risk factor in all the multivariate analyses, independently of the lung function parameter included in it, with the OR value ranging from 5.51 (95% CI 1.70–17.88), *p* = 0.005 to 4.94 (1.53–15.96), *p* = 0.008. The multivariate analysis showed that all the low lung function values analyzed, except FVC, were risk factors for wheezing at some time in their life (Figure 1).

### 3.2. Wheezing in the Past Year

A total of 14.8% of the children studied presented wheezing in the past year (Table 2). There were statistically significant differences (*p* = 0.013) in the prevalence of wheezing between the patients with BPD (24.6%) and no-BPD (10.4%). The univariate analysis showed that the presence of wheezing in the past year was associated with the FEF25-75 z-score (−1.47 ± 0.95 vs. −1.93 ± 0.91; *p* = 0.04) (Table 2).

**Figure 2:**
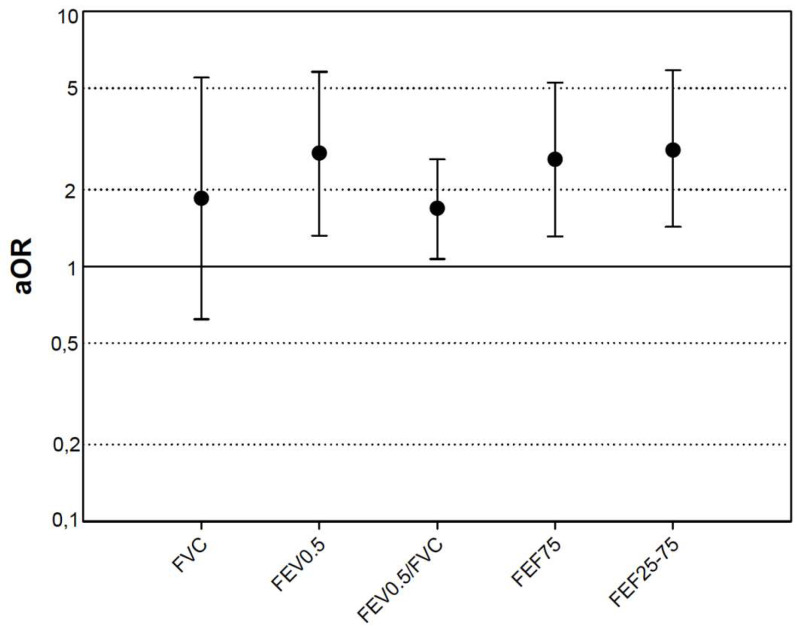
Risk of wheezing during the last year per z-score unit decrease of lung function. Adjusted odds ratios corrected for age, gender, daycare attendance, gestational age, birth weight (z-score), bronchopulmonary dysplasia, and allergic mother.

The multivariate analysis showed that all the lung function measurements analyzed, except FVC, were risk factors for wheezing in the past year (Figure 2), but suffering from BPD, although it showed a clear trend, was no longer a risk factor for wheezing in the past year (OR 2.46 (95% CI 1.07–5.63), *p* = 0.03 for the model that included FVC, but OR 3.16 (95% CI 0.89; 11.18) *p* = 0.074 in the model that included FEV05/FVC). The mother having an allergy also constituted a consistent risk factor in all the multivariate analyses, independently of the lung function parameter included therein, with the OR ranging from 7.66 (95% CI 1.67–35.04), *p* = 0.009 to 5.61 (1.39–22.67), *p* = 0.015.

### 3.3. Number of Wheezing Episodes

On the other hand, the number of wheezing episodes referred by the parents was significantly greater in those children who still presented wheezing in the past year (2.19 ± 0.6 vs. 0.41 ± 0.51; *p* < 0.001). However, the multivariate analysis showed that the number of exacerbations was related with all of the lung function parameters analyzed, except FVC, and having an allergic mother (Table 3), but attending day-care in the first year of life was not (β coef.: 0.18; 95% CI -0.11 to 0.47; *p* = 0.227).

### 3.4. Rehospitalisation Due to Respiratory Causes

The number of patients admitted to hospital for respiratory reasons was 27/140, which amounted to 19.29% (in two cases we do not have the data as the patients left the Region of Murcia). Of the children diagnosed with BPD, 20.9% were admitted to hospital on at least one occasion, compared to 16.7% of the preterm children without BPD (*p* = 0.534). No relationship was found between the variables studied and hospital admission or not due to respiratory causes. However, the multivariate analysis showed that a FEV0.5/FVC value lower than the lower limit of normality, was a risk factor for hospital admission (OR: 3.07; 95% CI 1.02 to 9.25; *p* = 0.046)

## 4. Discussion

In this study on the prevalence of wheezing in pre-school age children who had been born preterm, with or without BPD, almost half of them presented wheeze ever; however, only 21 out of 142 (14.8%) still presented wheeze in the past year, with the prevalence in children with BPD being slightly more than double as compared to those without BPD (18.2% vs. 9.2%). This prevalence is somewhat lower than the data published from the EPICURE study [6] on wheezing at 11 years of age in former very preterm children who, in the past year, 21% still had wheeze; although an additional 25% were diagnosed with asthma or were under treatment with asthma medication for respiratory symptoms. The children in that study had a mean age of 10.9 ± 0.38 years, significantly older than those in our sample (5.37 ± 1.64 years) and the percentage of patients with BPD was also higher in the EPICURE study (71% vs. 62%). In children between three and five years of age, the prevalence of wheezing was reported as 32% and 39%, respectively, in BDP and non-BDP patients (*p* = 0.51) [17]; however, the children in that study were born between 1998 and 2001, and our cohort were born between 2010 and 2016, which may explain the difference in prevalence. One study carried out in Germany [18], in a group of nine-year-old children who had been born preterm with a very similar gestational age to that of the present study, found a prevalence of wheezing of 14% and 4% in BDP and non-BDP patients, respectively, albeit without statistical significance, although this was probably due to the reduced size of the sample, which included only 28 patients in each group. It also corresponded to children born between 1994 and 2002; much earlier than our sample.

The respiratory morbidity of these former preterm infants is not only frequent, but also shows a potential severity, since almost 20% have been admitted to hospital, at least once in their lives, due to respiratory causes. We have found no significant differences in hospital admissions among those children diagnosed with BPD (20.9%) compared to those without (16.7%). The survey carried out by Vrijlandt et al. [17] referred to hospital admissions only in the first three months of life and found differences between BPD and non-BPD patients (54% vs. 14%; *p* < 0.001). The figures of rehospitalization in our study are, likewise, lower than those published by vom Hobe et al. [18] (36% and 21%, respectively). It is possible that the birth year of the children in each of the studies (the present one is later), as well as the use of palivizumab, systematic in our case and which is not reported in these two studies, can explain the lower number of patients admitted to hospital in our sample. Independently of the presence of BPD, in the total group, we found no association between hospital admittance and wheezing in the past year (18.9% vs. 21.6%; *p* = 0.706); although it almost reaches significance if we consider ever wheezing as opposed to never (64.9% vs. 35.1%; *p* = 0.06), probably as a consequence of those admitted due to bronchiolitis in the first year of life.

We have described for the first time, to the best of our knowledge, that infant lung function, essentially the obstructive pattern (FEV0.5/FVC) is a risk factor for respiratory morbidity: ever wheezing (Figure 1), wheezing in the past year (Figure 2) and also rehospitalization due to respiratory causes (FEV0.5/FVC < LLN: OR = 3.07; 95% CI 1.02 to 9.25; *p* = 0.046) in schoolchildren who were born preterm. It has been described that lung function in preterm children is lower with respect to their counterparts born at term, already in the infant stage [3] and that that alteration persists into school age [6] and even into adulthood [1]. Moreover, the preterm children who suffer BPD have a worse lung function than those born at term, during infancy [3,4], and even than the non-BPD preterm newborns [19], and this lost lung function, with regard to the healthy population, persists until school age [5,6] and even into adulthood [7,8]. The association of respiratory morbidity and alterations in lung function present at the moment of the survey or evaluation [7] has likewise been described, although not in all studies [2,6,18]. This would suggest that it is not only a structural reason which explains the respiratory morbidity, and that there must be other yet to be identified factors. For instance, in our case having an allergic mother is an independent risk factor for wheezing, although not for rehospitalization, which contradicts other publications that showed that family atopy is not a risk factor for wheezing among preterm children [2].

This study presents several limitations: firstly, the assessment of wheezing was carried out by means of a questionnaire and, although widely used throughout the world, this technique is always more subject to bias than if the assessment had been prospective. However, we believe that this bias cannot occur in the case of rehospitalization, given that the method used is the regional registry in which the clinical records of each patients are kept. Another limitation of the study is that no study of atopy had been performed in the patients; even more so if we consider that a relationship has been observed between maternal atopy and risk of wheeze. We do not really know how this information may have affected the results modifying the relationship between early lung function and subsequent wheezing, but it is probable that—as occurs with maternal allergy—this factor could have been independent of the best or worst early lung function. It would probably have modified the relationship between allergy in the mother and the higher prevalence of wheezing.

## 5. Conclusions

In conclusion, and apart from corroborating prior studies regarding the high prevalence of respiratory morbidity among children who were preterm newborns, we have found that the limitation in lung function at an infant age is a predictive factor both for subsequent wheezing and also for rehospitalisation.

## Figures and Tables

**Table 1:** Demographic characteristics, risk factors and lung function values and their differences between the children with or without ever wheezing.

	Total (142)	Wheezing: No (72)	Wheezing: Yes (70)	*p* *
Age (years)	5.37 (1.64)	5.22 (1.65)	5.53 (1.63)	0.27
Number of episodes	--	--	1.37 (0.65)	--
Gestational age (weeks)	27.7 (1.94)	27.76 (2.04)	27.77 (1.84)	0.98
Birthweight (g)	1034.3 (309)	1021.4 (287.6)	1047.6 (331.1)	0.62
Birthweight (z-score)	−0.33 (0.73)	−0.37 (0.79)	−0.28 (0.65)	0.47
Corrected age at test (months)	25.8 (15.7)	24.2 (16.4)	27.4 (14.8)	0.23
BPD	88 (62.0%)	38 (52.8%)	50 (71.4%)	0.022
Invasive ventilation	95 (66.9%)	48 (66.7%)	47 (67.1)	0.95
Day-care attendance in the first year	97(68.3%)	46 (63.9%)	51 (72.9%)	0.25
Male sex	84 (59.2%)	44 (61.1%)	40 (57.1%)	0.63
Smoking during pregnancy	27 (19.3%)	11 (15.5%)	16 (23.2%)	0.25
FVC (z-score)	−1.12 (0.57)	−1.10 (0.63)	−1.14 (0.51)	0.72
FEV_0.5_ (z-score)	−2.09 (0.87)	−1.94 (0.93)	−2.24 (0.79)	0.044
FEV_0.5_/FVC (z-score)	−0.45 (1.29)	−0.27 (1.36)	−0.62 (1.20)	0.11
FEF_75_ (z-score)	−1.47 (0.91)	−1.34 (0.93)	−1.61 (0.87)	0.07
FEF_25-75_ (z-score)	−1.54 (0.95)	−1.35 (0.97)	−1.73 (0.90)	0.02
Allergic mother	21(15.2%)	5 (7%)	16 (23.9%)	0.008
Rehospitalisation due to respiratory causes	37 (18.4%)	13 (13.3%)	24 (23.3%)	0.06

Indicates number of cases (percentage) or mean (SD). * Difference between the proportion of children with wheezing according to each risk factor.

**Table 2:** Demographic characteristics, risk factors and lung function values and their differences between the children with or without wheezing in the last year.

	Total (142)	Wheezing in Last Year: No (121)	Wheezing in Last Year: Yes (21)	*p* *
Age (years)	5.37 (1.64)	5.47 (1.66)	4.81 (1.47)	0.09
Number of episodes	0.68 (0.82)	0.41 (0.51)	2.19 (0.6)	<0.001
Gestational age	27.77 (1.94)	27.75 (1.94)	27.86 (1.98)	0.82
Birthweight (g)	1034.3 (309)	1014.3 (295)	1149.9 (366)	0.06
Birthweight (z-score)	−0.33 (0.73)	−0.37 (0.74)	-0.07 (0.62)	0.07
Corrected age at test (sem.)	25.8 (15.7)	25.6 (16.4)	26.7 (11.1)	0.77
BPD (yes)	88 (62%)	72 (59.5%)	16 (76.2%)	0.15
Invasive ventilation (yes)	95 (66.9%)	15 (71.4%)	80 (66.1%)	0.80
Day-care attendance in first year	71 (72.5%)	54 (68.9%)	17 (89.5%)	0.06
Male sex	62 (63.3%)	52 (65.8%)	10 (52.6%)	0.28
Smoking during pregnancy	15 (15.8%)	11 (14.5%)	4 (21.1%)	0.49
FVC (z-score)	−1.12 (0.57)	−1.11 (0.60)	−1.18 (0.39)	0.6
FEV_0.5_ (z-score)	−2.09 (0.87)	−2.04 (0.89)	−2.36 (0.72)	0.12
FEV_0.5_/FCV (z-score)	-0.45 (1.29)	−0.37 (1.30)	−0.89 (1.15)	0.09
FEF_75_ (z-score)	−1.47 (0.91)	−1.42 (0.90)	−1.79 (0.91)	0.08
FEF_25-75_ (z-score)	−1.54 (0.95)	−1.47 (0.95)	−1.93 (0.91)	0.04
Allergic mother (yes)	15 (16.3)	11 (14.9%)	4 (22.2%)	0.48
Rehospitalisation due to respiratory causes	36 (18.4%)	29 (17.9%)	8 (20.5%)	0.71

Indicates number of cases (percentage) or mean media (SD). * Difference between the proportion of children with wheezing according to each risk factor.

**Table 3:** List of number of episodes, referred by the parents, with different independent variables.

Variable	ß Coef. (95% CI)	*p*
FVC (z-score)	−0.073 (−0.33; 0.18)	0.575
FEV_0.5_ (z-score)	−0.20 (−0.37; −0.04)	0.015
FEV_0.5_/FVC (z-score)	−0.13 (−0.23; −0.03)	0.015
FEF_75_ (z-score)	−0.21 (−0.36; −0.06)	0.007
FEF_25-75_ (z-score)	−0.22 (−0.36; −0.07)	0.003
